# Contrasting contributions of movement onset and duration to self‐evaluation of sensorimotor timing performance

**DOI:** 10.1111/ejn.15378

**Published:** 2021-07-13

**Authors:** Ljubica Jovanovic, Joan López‐Moliner, Pascal Mamassian

**Affiliations:** ^1^ Laboratoire des systèmes perceptifs, Département d'études cognitives, École normale supérieure PSL University, CNRS Paris France; ^2^ Vision and Control of Action (VISCA) Group, Department of Cognition, Development and Psychology of Education, Institut de Neurociències Universitat de Barcelona Barcelona Catalonia Spain; ^3^ School of Psychology University of Nottingham Nottingham UK

**Keywords:** duration perception, metacognition, movement planning, sensorimotor synchronization

## Abstract

Movement execution is not always optimal. Understanding how humans evaluate their own motor decisions can give us insights into their suboptimality. Here, we investigated how humans time the action of synchronizing an arm movement with a predictable visual event and how well they can evaluate the outcome of this action. On each trial, participants had to decide when to start (reaction time) and for how long to move (movement duration) to reach a target on time. After each trial, participants judged the confidence they had that their performance on that trial was better than average. We found that participants mostly varied their reaction time, keeping the average movement duration short and relatively constant across conditions. Interestingly, confidence judgements reflected deviations from the planned reaction time and were not related to planned movement duration. In two other experiments, we replicated these results in conditions where the contribution of sensory uncertainty was reduced. In contrast to confidence judgements, when asked to make an explicit estimation of their temporal error, participants' estimates were related in a similar manner to both reaction time and movement duration. In summary, humans control the timing of their actions primarily by adjusting the delay to initiate the action, and they estimate their confidence in their action from the difference between the planned and executed movement onset. Our results highlight the critical role of the internal model for the self‐evaluation of one's motor performance.

## Graphical abstract

We investigated how humans time the action of synchronizing an arm movement with a predictable visual event and how well they can evaluate the outcome of this action. Participants mostly varied their movement onset, keeping the average movement duration short and relatively constant across conditions. Results showed that confidence judgements reflected deviations from the planned reaction time, and not planned movement duration, highlighting the critical role of the internal model for the self‐evaluation of one's motor performance.

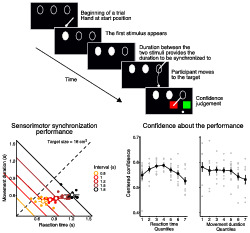

**Abbreviation**
MLE: maximum likelihood estimation

## 1 INTRODUCTION

Movement planning and execution are underconstrained problems. For example, a simple reaching movement can be accomplished with an infinite number of trajectories and durations (Engelbrecht, 2001). In spite of this large uncertainty, humans plan and execute actions in a manner that maximizes their gain in visuo‐motor tasks. More specifically, it has been shown that humans take account their sensory and motor uncertainty (Battaglia & Schrater, 2007; Faisal & Wolpert, 2009) as well as biomechanical costs (Cos et al., 2011, 2012, 2014; Soechting & Lacquaniti, 1981). Moreover, they integrate information about uncertainty with externally imposed gains in space (Dean et al., 2007; Trommershäuser et al., 2003) and time (Hudson et al., 2008), resulting in optimal movement execution that maximizes the gain in a specific context.

Even though humans aim to maximize their gain in sensorimotor tasks, movement execution is not always optimal (Jarvstad et al., 2014; Mamassian, 2008; Wang et al., 2016; Wu et al., 2009; Zhang et al., 2010; for a review, see Maloney & Zhang, 2010). For example, in an anticipatory motor task, participants were asked to estimate when the last stimulus, in a sequence of three, would appear and press a key to synchronize their keypress with the timing of that stimulus (Mamassian, 2008). Participants were overconfident in their performance in that they underestimated the hitting variability of their action or they overestimated the value of rewards. Furthermore, executing movements optimally is particularly challenging when movement is continuous (closed‐loop) or when context is volatile. For example, allocation of a fixed time to the two targets leads to suboptimal and biased performance (Wu et al., 2009), and learning complex distributions or switching between contexts is slow and difficult (Acerbi et al., 2012; Karniel & Mussa‐Ivaldi, 2002; Narain et al., 2013; Roach et al., 2017).

Estimating the correctness of one's own perceptual or motor decisions can give us some insights on the reasons why certain pattern of behaviour is selected. Deciding on the correctness of one's own performance is called a confidence judgement. Studies of confidence have provided good evidence that humans are able to reliably estimate the precision of their own performance in visual perception tasks, such as spatial frequency, orientation or motion direction discrimination (Barthelmé & Mamassian, 2009, 2010; de Gardelle & Mamassian, 2014; Yeung & Summerfield, 2012). Furthermore, confidence judgements on a sensory task are also informed by the motor system, and disrupting response motor representations in premotor cortex selectively disrupted confidence judgements (Fleming et al., 2015). The ability to estimate confidence in executed actions has been far less researched. Recent work investigating temporal error monitoring suggests that humans can explicitly estimate the degree and sign of their temporal errors (Akdoğan & Balci, 2017; Kononowicz et al., 2019), but our understanding of what information is utilized for these judgements of confidence is still limited.

In the work presented here, we investigated how humans time an action when asked to synchronize their arm movement with temporally predictable visual stimuli. We asked two questions. First, we investigated how participants trade off reaction time and movement duration in relation to different sources of uncertainty. On each trial, participants had to estimate the interval between the first two stimuli and then decide when to start (reaction time) and for how long to move (movement duration) to reach the target on time. We manipulated target size and orientation in an attempt to affect the movement duration needed to perform the task correctly. More specifically, the smaller the target, the higher the spatial precision required, and the longer the movement duration (Fitts, 1954). Furthermore, movement end points have a distribution that is anisotropic, with a larger variability along the principle axis of movement (Apker et al., 2010; Rossetti, 1998; van Beers et al., 2004). This manipulation of target size and orientation thus allowed us to investigate whether timing an action is sensitive to and compensates for temporal requirements of movement execution related to spatial difficulty. Furthermore, we varied the temporal interval to be reproduced. Because shorter intervals are encoded with better precision (Gibbon, 1977; Jazayeri & Shadlen, 2010), this manipulation of temporal interval allowed us to investigate the trade‐off between spatial and temporal stimulus uncertainties. The second question we asked is whether humans can estimate the correctness of their own actions and whether accuracy and confidence in actions are affected by the same factors. Perception of time and its reproduction rely on different brain structures (Bueti et al., 2008), and thus, performance and confidence can be disrupted independently (Fleming et al., 2015; Rahnev et al., 2011). Although there is evidence that humans can monitor their temporal reproduction errors (Akdoğan & Balci, 2017; Kononowicz et al., 2019), it is not clear whether temporal motor variability is always estimated correctly (Hudson et al., 2008; Mamassian, 2008). Furthermore, a study investigating temporal error monitoring in a temporal production task showed that power in the alpha range (8–14 Hz) *after* the reproduction was related to both reproduction duration and temporal error estimation (Kononowicz & van Wassenhove, 2019). Shorter reproduction times were followed by stronger synchronization in the alpha band. Stronger synchronization was also found for trials that were estimated to be ‘too short,’ although the mechanism of self‐evaluation yielding changes in oscillations in alpha band is still elusive. Interestingly, there was no reliable neuronal correlate of the meta‐cognitive process *before* the end of the reproduction. This pattern of results suggests that only once the duration is produced, the estimated temporal error is assessed. However, humans can change their mind during motor execution (Resulaj et al., 2009) and correct their movement to account for the incoming information with a delay of about 100 ms (Brenner & Smeets, 1997). Here, we asked participants to perform a sensorimotor task that allowed us to disentangle the two temporal components of their performance: reaction time and movement duration. While both reaction time and movement duration will inevitably affect performance, it is an open question whether confidence about performance depends on both temporal components of the movement equally.

## 2 MAIN EXPERIMENT

### 2.1 Methods

#### 2.1.1 Participants

Ten human participants (five males, two left handed, mean age 24.8) took part in the experiment after giving their informed consent. All participants had normal or corrected to normal vision. Sample size was determined based on previous studies (e.g., Barthelmé & Mamassian, 2009; Mamassian, 2008; Trommershäuser et al., 2003). The study was conducted at University of Barcelona and was part of a programme that was approved by the University of Barcelona's ethical committee.

#### 2.1.2 Apparatus

Participants sat in front of a drawing tablet (Calcomp Drawing Tablet III 24240) that recorded movements of a hand‐held stylus. Stimuli were projected from above by a Mitsubishi SD220U ceiling projector onto a horizontal back‐projection screen positioned 40 cm above the tablet. Images were projected at a frame rate of 85 Hz and a resolution of 1024 by 768 pixels (61 × 45 cm). A half‐silvered mirror midway between the back‐projection screen and the tablet reflected the images shown on the visual display giving participants the illusion that the display was in the same plane as the tablet. Lights between the mirror and the tablet allowed participants to see the stylus in their hand. A custom programme written in C and based on OpenGL controlled the presentation of the stimuli and registered the position of the stylus at 125 Hz. The software ran on a Macintosh Pro 2.6‐GHz Quad‐Core computer. The set‐up was calibrated by aligning the position of the stylus with dots appearing on the screen, enabling us to present visual stimuli at any desired position of the tablet.

#### 2.1.3 Stimuli

**FIGURE 1:**
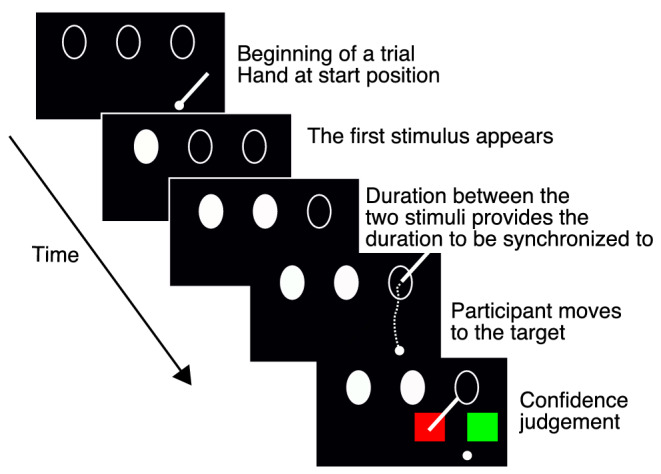
Schematic representation of one trial (plan view). A trial starts when the participant's hand is at the starting position, and the three outline stimuli are presented on a horizontal plane. The first two outlines fill in white sequentially with a specific delay for that trial. Participants had to reach the third target with a delay relative to the second outline that matched the delay between the first two. After movement execution, two squares (green and red) are presented below the target. Participants estimate the confidence in the accuracy of their performance by placing their hand within the red (performance worse than average) or green square (performance better than average)

Stimuli were three white ellipses presented sequentially on the horizontal plane on a black background. The centre of the ellipse was located 25 cm from the home position. We systematically varied size, orientation and temporal interval between the sequential presentation of the stimuli. The length of the major and minor axes of the ellipses was varied in four equal linear steps, from 0.8 to 3.2 and from 0.4 to 1.6 cm, respectively. We tested two orientations, with the major axis being either perpendicular or aligned with the arm movement (as in the example shown in Figure 1). In the rest of the report, we refer to these two orientations as horizontal and vertical, respectively. Across trials, the temporal intervals were varied in five steps that were approximately logarithmically equally spaced (0.8, 1, 1.2, 1.5 and 1.8 s). All conditions (temporal intervals, target sizes and orientation) were intermixed within blocks.

#### 2.1.4 Procedure

Participants were asked to synchronize the end of their hand movements with a temporally predictable visual stimulus presented on a horizontal plane. After the consecutive presentation of the first two stimuli, participants moved their dominant hand in order to point to a target third stimulus. The timing of the target could be inferred from the interval delay between the first two stimuli. In other words, participants had to estimate the duration between the first and the second stimulus presentations. Then, they had to plan and execute the movement so as to reach the third stimulus at the moment this stimulus was presented. They could anticipate that time because the temporal interval between the first two stimuli equalled the temporal interval between the second stimulus and the third.

Each trial started with participants placing their hand holding a pen at the initial position (see Figure 1), and the outlines of three ellipses were presented, all with same size and orientation for that trial. First the leftmost and then the middle ellipses were filled in white. Participants were asked to synchronize their movement with the predictable sequence given by interval between the first two ellipses and to land at the target third ellipse at the time it would have turned white. The target ellipse never actually became white, so no temporal feedback was provided during the experiment. After each set of 50 trials, participants were informed about their cumulative performance on the set. For the two left‐handed participants, the display was reversed, so that they could perform the experiment naturally with their dominant hand.

For each trial, after the movement was executed, participants estimated their confidence about their performance for that trial. The instructions to the participants were that they should estimate both the spatial and the temporal aspects of their own performance, relative to their average performance across previous trials. They were asked to provide a binary confidence estimate, by answering whether on that trial their performance was better or worse than the average performance on all previous trials. These instructions were to convey to the participants that they should try to perform well on both spatial and temporal dimensions (and adjust their movement duration and end‐point variability to different target sizes). However, because participants could see their hand and the target, the spatial component of their performance was obvious. Five participants completed 50 trials per condition, and five participants completed 40 trials per condition (yielding 2000 and 1600 trials per participants, respectively). The experiment lasted approximately 5 h, and it was done in four or five sessions.

Before starting the experimental session, participants completed 80 training trials with feedback. During this practice, participants either heard a tone if they performed correctly (reached the target within ±15% of the target interval) or otherwise saw a horizontal bar of a length that corresponded to the magnitude and direction of temporal error (if the bar was on the left from the target, the target was reached too early, and if on the right, too late).

#### 2.1.5 Data analysis

The analysis of the data was conducted in R Studio environment, using packages ‘lme4’ (Bates et al., 2015) and ‘car’ (Fox & Weisberg, 2011) for mixed effect regression analysis. Position data were smoothed with a Butterworth filter in order to filter out high‐frequency noise from the data (cut‐off at 8 Hz).

Reaction times were calculated relative to the onset of the second stimulus, by using the Algorithm A defined in Teasdale et al. (1993). Movement offset was defined as the time when hand velocity was less than 1.2 cm/s (de la Malla & López‐Moliner, 2012). Movement duration was calculated as the temporal interval between the reaction time and movement offset.

We cleaned the data by removing trials that had extreme reaction times (larger than 2.5 s) and movement duration (larger than 2 s). Less than 5% of the data were excluded from the analysis.

For statistical analyses of the data, we used linear mixed effect models, because they incorporate random effects (here on the level of subject) and flexibly represent the covariance structure originating from the grouping of the data (Laird & Ware, 1982; Pinheiro & Bates, 2006). Furthermore, these analyses do not require averaging of the data and take the whole data set into account (Baayen et al., 2008; Winter, 2013). In addition, *lmer* function implemented in R (Bates et al., 2015) was shown to produce sensible maximum likelihood estimation (MLE) estimates from unbalanced data as well, which is important for our analyses, because we had to exclude some trials (movement duration and reaction times outside a reasonable range, spatial misses for the confidence analysis) (Pinheiro & Bates, 2006). When using generalized linear mixed‐effect models for testing models with a binary dependent variable, we used the *logit* link in the *glmer* function. Details of the model structure for each of the models are described in the results sections.

## 3 RESULTS AND DISCUSSION

### 3.1 Spatial accuracy is affected by the size and orientation of the target

**FIGURE 2:**
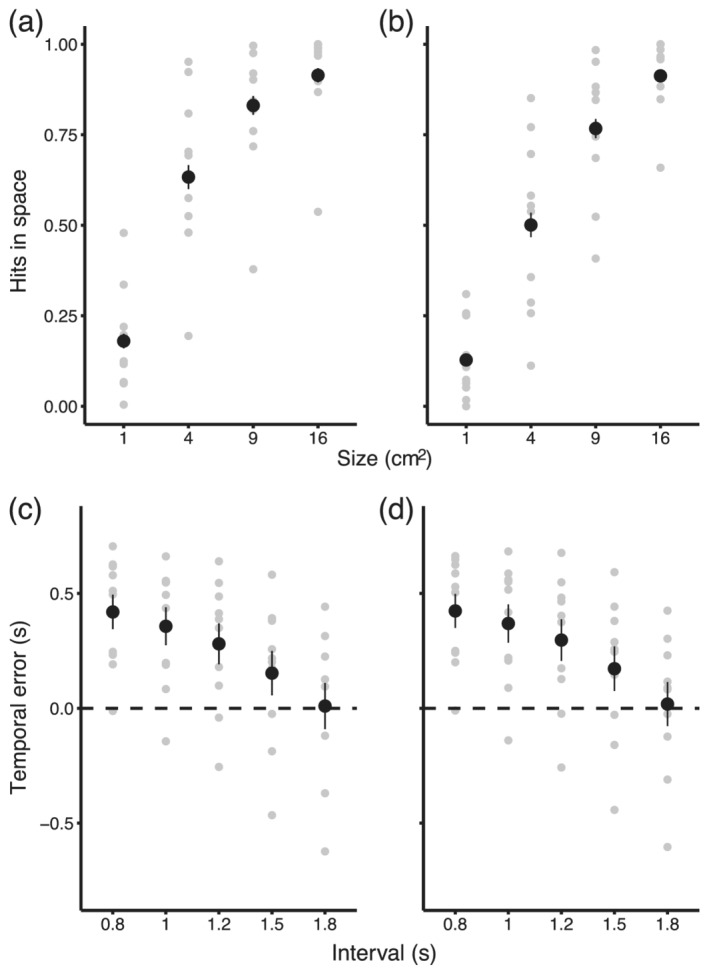
Spatial and temporal performance. Proportion of spatial hits against stimulus size for horizontally (a) and vertically (b) oriented targets. Data are averaged across different intervals. Performance increased with target size, reaching ceiling performance for the largest target size. Temporal errors against presented interval for horizontally (c) and vertically (d) oriented targets. Temporal errors are the differences between hitting times (temporal interval between the second target and the moment when participants ended their movement) and interval between the first two stimuli. Positive errors indicate that participants responded too late. Temporal errors are averaged across target sizes. Temporal errors decreased with the interval, reaching bias‐free performance for the longest interval on average. In all panels, error bars represent standard error of the mean across participants and grey symbols individual data

We quantified performance on the spatial aspect of the task by calculating the proportion of trials for which participants landed their movement inside the target stimuli (spatial hit), separately for each condition. Hitting accuracy was affected by the size of the target and orientation but not by the interval duration participants were synchronizing to (Figure 2a,b). To quantify this effect, we fitted the data with a generalized linear mixed‐effect model. The logarithm of presented interval, the target size and its orientation were within‐subject fixed effects. Interval and size were continuous variables, and orientation was treated as a categorical factor with two levels. We also included the interaction between size and orientation in the analysis. The dependent variable was hit/miss on a given trial, and the random structure consisted of a random intercept and a slope for size for each subject to account for additional variability. We observed a significant effect of target size (Wald Chi‐square(1) = 161.307, *p* < .01) and orientation (Wald Chi‐square(1) = 35.27, *p* < .01). Participants were more likely to perform correctly if the target was larger (slope = 4.06 rate of change of odds for answering correctly with increments of size exp(4) = 54, SE = 0.32, *p* < .01). Probability of correct response was greater for vertical than horizontal targets (*b* = −0.57 [exp(b) = 0.57], SE = 0.09, *p* < .01). There was no effect of temporal interval (Wald Chi‐square(1) = 0.6, *p* = .430) or significant interactions between factors.

### 3.2 Synchronization to the temporal sequence is affected by interval and target orientation

We quantified performance on the synchronization task by calculating a temporal error between hitting time and presented temporal interval. Hitting time was defined as the temporal interval between the presentation of the second target and the moment when participant landed on the tablet. Positive errors indicate that the interval between the second stimulus and end of the arm movement was longer than the interval between the first two stimuli; that is, the target was reached too late. Negative errors indicate that the target was reached too early. In Figure 2c,d, temporal errors are plotted against presented interval between the first two stimuli. We analysed the results with a linear mixed‐effect model, with the logarithm of presented interval, target size and orientation as within‐subject fixed effects. The dependent variable was the signed temporal error, and as random effects, we allowed intercepts and slopes of all predictors to vary for each subject to account for additional variability. The analysis showed that temporal error decreased significantly with the logarithm of presented interval (Wald Chi‐square(1) = 64.8, *p* < .01). Temporal error decreased when the logarithm of presented interval increased (slope = −1.23, SE = 0.157, *t* = −7.863, *p* < .01). Orientation had a small but significant effect on temporal error (Wald Chi‐square(1) = 4.9, *p* < .05), and temporal error was slightly larger for vertically oriented targets (slope = 0.014, SE = 0.007, *t* = 2.2, *p* < .05). There was no effect of target size (Wald Chi‐square(1) = 3.2, *p* = .08), although there was an interaction between the presented interval and the size (Wald Chi‐square(1) = 22.7, *p* < .01; slope = 0.14, SE = 0.03, *t* = 4.764, *p* < .01).

### 3.3 Confidence about performance is affected by interval and target size

**FIGURE 3:**
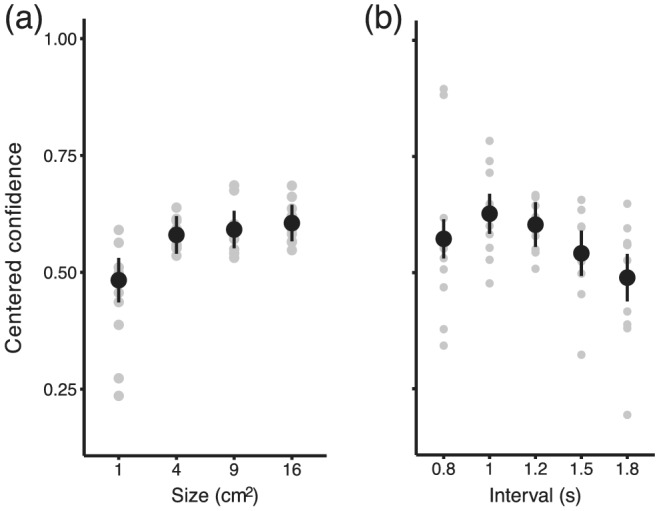
Confidence judgements in Experiment 1. (a) The proportion of trials estimated to have better than average performance is plotted against target size. Confidence of participants about their performance increased with the size of the target. (b) The proportion of trials estimated to have better than average performance is plotted against interval duration. Confidence about one's performance was smaller for longer temporal intervals, even though performance was more accurate for these longer intervals. In both of these plots, confidence is restricted to trials where participants correctly responded within the target, so that the lower confidence for small sizes cannot be attributed to a visible spatial error. In all panels, error bars represent standard error of the mean across participants and grey symbols individual data

On each trial, we asked participants to estimate their confidence about their movement performance. More specifically, participants had to estimate if on a particular trial they were better or worse than on the average of all previous trials. In other words, they had to distribute high and low confidence estimations so that their counts would be roughly equal at the end of the experiment. These binary responses are summarized in Figure 3 and plotted against presented interval duration. Because participants could see their hand, they had feedback about their performance on the spatial component of the task. For that reason, for the analysis of confidence judgements, we used only trials in which the hand landed within the target (hits in space). Keeping the confidence question on both space and time was nonetheless important to motivate participants to make accurate movements in both space and time. For completeness, temporal errors and confidence judgements for spatial hits and misses are shown in Figures S1 and S2. We quantified the effects by means of a generalized linear mixed‐effect model, with binary confidence judgement as the dependent variable. We included the logarithm of interval, target size and orientation as predictors. Also, to account for the non‐linearity of the effect of interval (see Figure 3), we included a quadratic term (interval^2^). The random structure consisted of random intercepts and slopes for the interval and size predictors on the subject level. Significant predictors of confidence judgements were logarithm of the target size (Wald Chi‐square(1) = 7.3, *p* < .01) and the squared interval (Wald Chi‐square(1) = 46.95, *p* < .01). Odds of being confident in performance on a given trial were increasing with the logarithm of target size (slope = 0.46, exp(slope) = 1.5, SE = 0.170, *z* = 2.70, *p* < .01). In addition, we observed a non‐linear relationship between interval and confidence (slope = −2.17, SE = 0.4, *z* = −6.852, *p* < .01).

### 3.4 How do participants trade off reaction time and movement duration?

**FIGURE 4:**
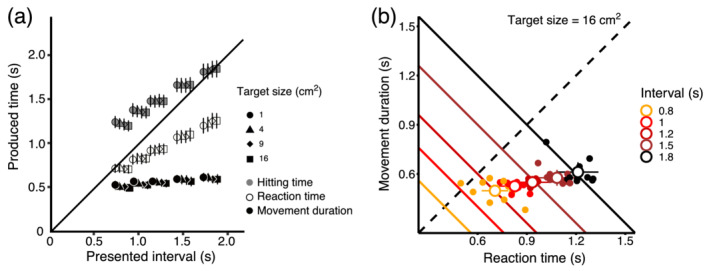
Contributions of reaction times and movement durations to hitting times. (a) Reaction time, movement duration and hitting time are plotted against intervals for different target sizes. If participants were accurate, their hitting time would fall on the diagonal. Participants reached the target too late on average (hitting time was too long), except for the longest presented interval. Results are averaged across participants and across the two target orientations. (b) Average movement durations are plotted against reaction time for the five different intervals (darker shades of red indicate longer intervals). Only data for the largest target size are shown (the data for the other target sizes are similar). Using the same colour code as for the data, diagonal lines indicate the trade‐off between reaction time and movement duration that are compatible with the presented intervals. On average, participants tended to delay the movement onset and make brief movements (data are below the identity dashed line where reaction times equalled movement durations). Most of the variability between conditions was due to different reaction times. Error bars indicate standard error of the mean across participants, and filled symbols show individual data (centred to participant's mean, Loftus & Masson, 1994)

We now analyse separately reaction time (the moment when participants started moving) and movement duration (interval between the movement onset and the end of the movement). Hitting time, the moment participants landed on the target, is the sum of reaction time and movement duration. Because the effects reported here are similar across target orientations, we pool the data across these two orientations. Reaction time and movement duration as a function of presented interval and target size are summarized in Figure 4a. As presented interval increased, hitting time naturally increased, but reaction time and movement duration did not evolve at the same rate. As the hitting times increased, participants displayed a general tendency to delay their reaction time while keeping movement durations relatively brief and constant. This tendency can be clearly seen in Figure 4b, where the average movement duration is plotted against the average reaction time, for different interval durations and a single target size (16 cm^2^). Diagonal lines indicate valid trade‐offs between reaction times and movement durations for the different interval durations tested (colour coded from light orange to black for interval durations between 0.8 and 1.8 s). For example, in order to reach the target perfectly on time in the 0.8‐s interval condition, participants could trade off the reaction time and movement duration differently as long as they belong to the orange diagonal line, so that their hitting time (sum of the movement duration and the reaction time) was indeed 0.8 s. Instead, participants arrived to the target too late (individual participant data shown as orange dots, all of them lying above the diagonal). Furthermore, as the target interval duration increased, participants mostly delayed their reaction time, thereby creating a horizontal shift of the mean trade‐off between reaction time and movement duration. Average distributions for each of the conditions in Experiment 1 are shown in Figures S3 and S4.

To assess whether the way participants trade off reaction time and movement duration was affected by conditions in our experiment, we fitted a linear mixed‐effect model with logarithm of ratio between reaction time and movement duration as dependent variable, logarithm of interval, size and their interaction as continuous predictors and orientation as factor. The ratio between reaction time and movement duration is a relative measure of how participants trade off reaction time and movement duration on a particular trial. Random structure consisted of intercept and slope for interval (maximal structure allowed by the data), to account for additional variability between participants. The results showed an effect of presented interval (Wald Chi‐square(1) = 18.4, *p* < .01), size (Wald Chi‐square(1) = 63.11, *p* < .01) and the interaction between the two predictors (Wald Chi‐square(1) = 14.32, *p* < .01). Orientation was not a significant predictor (Wald Chi‐square(1) = 0.120, *p* = .73). These results suggest that increasing both the interval (slope for logarithm of interval = 0.408, SE = 0.095, *t* = 4.29, *p* < .01) and the size (slope for logarithm of target size = 0.023, SE = 0.0031, *t* = 7.944, *p* < .01) of the target leads to an increase in reaction time relative to movement duration. Moreover, because we observed a significant positive interaction between the predictors, the effects of the interval are different for different target sizes, namely, the larger the size, the larger was the effect of interval on the trade‐off (slope = 0.070, SE = 0.019, *t* = 3.785, *p* < .01).

To further investigate how well people perceive, monitor and adjust movement duration while performing a movement, we checked whether participants took the relationship between movement duration and target size into account when they planned the movement. If they did, we would have expected differences in reaction time for different target sizes (small targets require longer movement durations, so reaction time should be shorter in these conditions). We observed an interaction between interval and target size on reaction time. Reaction time increased with interval duration (Wald Chi‐square(1) = 42.272, *p* < .01, *b* = 1.25, SE = 0.182, *t* = 6.875, *p* < .01), and the increase was larger for larger interval and larger targets (Wald Chi‐square(1) = 32.250, *p* < .01, *b* = 0.154, SE = 0.027, *t* = 5.584, *p* < .01). These findings suggest that participants did take into account target size when executing the movement, although this relationship depended on the interval duration.

### 3.5 Does confidence in performance depend on the way we perform an action?

After executing each timed movement to a predictable target, participants estimated whether that movement was better or worse than the average movements they had executed so far. We are interested in the factors that contribute to this motor confidence judgement, focusing in particular on reaction time and movement duration. We hypothesized that confidence was related to absolute deviations from a planned action. More specifically, participants may have assigned a low confidence to a trial that deviated strongly from the intended plan. Planned reaction times and movement duration can be simply estimated from the data by considering the median reaction time and movement duration for each condition (Dean et al., 2007). Deviations from the median values measure the difference between planned and performed movement on a particular trial. Therefore, for each participant, we calculated the deviations from the median reaction time and movement duration for each condition.

To test the hypothesis that deviations from planned reaction time and movement duration could be used for judgements of confidence in the performance on a given trial, we fitted the data with a generalized mixed‐effect model, with signed and squared deviations from median reaction time and movement duration as predictors and confidence judgements as the dependent variable. We included a random structure that consisted of random intercepts and slopes for interval and target size on the level of participant. We observed an effect of squared deviation from median reaction time (Wald Chi‐square(1) = 19.7, *p* < .01) and no significant linear trend (reaction time: Wald Chi‐square(1) = 1.55, *p* = .21; movement duration: Wald Chi‐square(1) = 2.67 *p* = .11) or squared movement duration deviation (Wald Chi‐square(1) = 1.9, *p* = .17). The greater the squared deviation from the median reaction time on a given trial, the smaller the probability for that trial to be chosen as ‘confident’ (*b* = −1.79, SE = 0.40, *z* = −4.439, *p* < .01).

Because participants completed a large number of trials, learning over the course of the experiment could alter both motor performance and confidence judgements. To account for these effects, we fitted individual performance over the course of the experiment with an exponential function, separately for each participant and variable (reaction time, movement duration and confidence estimation). The fitting procedure is detailed in the Supporting Information, and illustration of exponential fits is shown in Figure S5. The analyses below are performed on the data detrended for learning or fatigue effects, that is, on the residuals computed as the trial‐to‐trial difference between observed data and values predicted by the exponential fits.

**FIGURE 5:**
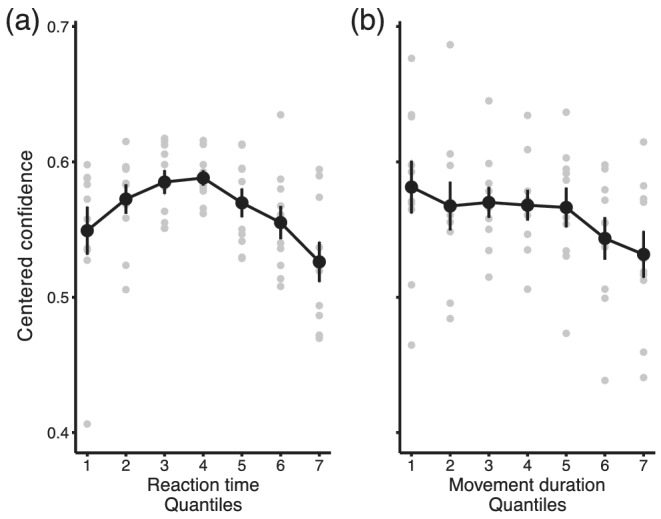
Relationship between confidence and reaction times and movement durations in Experiment 1. (a) Average proportion of responses ‘performance better than average’ (centred, Loftus & Masson, 1994) is plotted against signed deviation from median reaction time (quantiles). Confidence was greater when the reaction time on a given trial was closer to the median. (b) Average proportion of responses ‘performance better than average’ (centred) is plotted against signed deviations from median movement durations (quartiles). There was a linear decrease in confidence in the performance for longer movement durations. Error bars indicate standard errors of the mean across participants, and grey points show individual data. Deviations from median reaction times and movement durations were averaged across the seven quantiles of their distributions, for the purpose of data visualization

The analyses with detrended values of reaction time, movement duration and confidence confirmed the previous finding. Namely, squared deviations from the median reaction time were negatively related to confidence judgements (Wald Chi‐square(1) = 28.7, *p* < .01, *b* = −2.45, SE = 0.46, *z* = −5.356, *p* < .01). However, in addition to the effect of squared deviations from the response time, both linear predictors were significant, signed deviations from reaction time (Wald Chi‐square(1) = 5.9, *p* < .05) and movement duration (Wald Chi‐square(1) = 11.86, *p* < .01). The smaller the reaction time (*b* = −0.37, SE = 0.152, *z* = −2.437, *p* < .05) and movement duration relative to the median (*b* = −0.9, SE = 0.266, *z* = −3.444, *p* < .01), the greater the probability of ‘confident’ responses. In Figure 5, detrended confidence is plotted against signed deviations from median reaction times and movement durations (also detrended). For the purpose of data visualization only, the distributions of deviations were binned in seven equally sized bins, and confidence estimates were averaged for each bin.

## 4 SUPPORTING EXPERIMENTS

In the first experiment, we found evidence that humans use information about different components of the movement differently when asked to evaluate their performance. In particular, we found that confidence judgements took into account deviations from planned reaction time but not movement duration. To further investigate the relationship between self‐evaluation of performance and the two temporal components of the movement, we conducted two additional experiments.

The purpose of Experiment 2 was to address several aspects of the main experiment that could affect our conclusions, both at the level of the independent variables and the task. We introduced three modifications to our independent variables. First, in the main experiment, several temporal intervals were presented to participants, and both confidence and reaction time depended on temporal interval. In Experiment 2, participants were also asked to synchronize their arm movement with a predictable temporal sequence of visual stimuli as in the first experiment, but only one interval (1.5 s) was tested. Presenting a single temporal interval during the whole experiment excluded differences in sensory noise as a potential cue for confidence judgements, as well as differences in the median reaction times and corresponding deviations. Second, in the main experiment, reaction times were considerably longer than movement duration even for the smallest targets and so was their variability. In order to manipulate more strongly movement duration, we manipulated target distance in separate blocks in Experiment 2. Third, in order to further minimize the contribution of spatial uncertainty from the self‐evaluation of performance, target size was scaled with target distance (Fitts, 1954).

**FIGURE 6:**
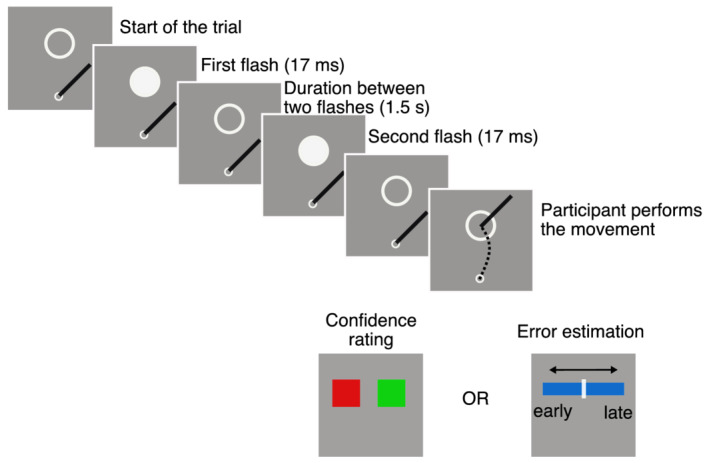
Schematic representation of experimental sequence in Experiment 2 (plan view). A trial begins when the participant's hand is at the starting position. A stimulus outline is presented above the starting position, and this outline is filled as a white disc two times, thereby producing two flashes separated by 1.5 s. Participants have to estimate the temporal interval between the two flashes and move their hand to the outline at a time after the second flash that equals this interval (1.5 s). After movement execution, participants are asked either to judge their confidence in their performance or to estimate their temporal error. In the confidence task, two squares (green and red) are presented below the target. Participants estimate the confidence in the accuracy of their performance by placing their hand within the red (performance worse than average) or green square (performance better than average). In the temporal error estimation task, participants place the cursor on a blue line to indicate their temporal error. Distance from the line midpoint indicates the magnitude, and the side (left or right from the midpoint) indicates the direction of the error (early or late)

We also introduced some changes in the task. In addition to our method of single stimuli to probe confidence, we chose another method of self‐evaluation similar to the one used in related work (Akdoğan & Balci, 2017; Charles & Yeung, 2019; Kononowicz et al., 2019). Therefore, we were able to compare two tasks. The first task was the same as the one in Experiment 1: after each trial, participants estimated the confidence in their sensorimotor performance, relative to all previous trials (more or less confident that they performed correctly). The second self‐evaluation task was to estimate the magnitude and sign of their temporal error on each trial, by means of a scale presented after the trial (Figure 6). Participants were asked to perform the two tasks in separate blocks.

The purpose of Experiment 3 was to reduce the contribution of movement duration in the estimation of motor confidence. We decided to remove movement duration altogether by asking participants to respond by pressing a key on a keyboard instead of moving their arm. When performing a keypress, temporal performance now depends almost entirely on reaction time, and this experiment allows us to compare our findings more directly with previous studies that used exclusively keypresses as a response method. As in Experiment 2, there were two self‐evaluation tasks, confidence judgement and error estimation.

### 4.1 Methods

#### 4.1.1 Participants

Eleven participants took part in both Experiments 2 and 3 (four males). All participants were naive to the purpose of the experiment and signed the written informed consent form. One participant did not comply to task demands (always responded ‘more confident’ in the confidence task), and their data were not further analysed.

#### 4.1.2 Stimuli and apparatus

The stimulus was a white circle, whose size varied as a function of its distance from the starting point (radius 0.5, 1.5 or 2.5 cm). The size of the target was adjusted to account for the scaling of the spatial end‐point variability with movement amplitude (Fitts, 1954; Soukoreff et al., 2011). The sizes of the targets presented at 20 and 35 cm from the participant were scaled according to modified Fitts' formula, where index of difficulty equals the binary logarithm of one plus quotient of target distance and size.

To present stimuli and record the movement trajectory, a Wacom Cintiq 27QHD Touch (2560 × 1440) running on 60‐Hz refresh rate was used. Participants used a Wacom stylus to move on the surface of the screen. Stimuli and data acquisition were controlled by MATLAB 2016b and PsychToolbox running on Mac Mini OS X.

#### 4.1.3 Procedure

Each participant completed both Experiments 2 and 3 that differed in the response methods. In Experiment 2, participants moved their hand to reach a distant target at a particular time, whereas in Experiment 3, they just had to reproduce an interval duration. After they performed this timed motor task, they were asked to make a self‐evaluation of their performance, either by reporting their confidence in their performance or by indicating an estimate of their temporal error.

The movement task of Experiment 2 was similar to that in Experiment 1, with a few differences. In different blocks, the target position relative to the participant was varied, and the target size was scaled with distance (Fitts, 1954). In addition, the position of the stimulus was jittered, to discourage stereotypical movements towards the target. Finally, instead of presenting three stimuli separated spatially, the stimulus remained at the same location within a trial. A trial started when participants placed the stylus inside the home position. At the beginning of the trial, an outline of the stimulus appeared. After 200 ms, the stimulus turned briefly (17 ms) white twice. The temporal interval between these two consecutive events was constant during the experiment (1.5 s). Participants had to move the stylus on the surface of the tablet to the outline with a delay relative to the second event that matched the temporal interval. An illustration of the stimulus sequence is shown in Figure 6.

In the interval reproduction task of Experiment 3, the experimental sequence was identical to that of the movement task, except that participants had to press a key instead of moving their hand. Participant's task was thus to synchronize the keypress with the temporal sequence given by the temporal interval between the two white discs. In addition, the stimulus position and its size were kept constant. As in the movement task of Experiment 2, in two separate blocks, participants estimated their confidence in their performance or their temporal error.

For both Experiments 2 and 3, participants were asked to evaluate their performance after each trial with one of two methods. In different conditions and separate blocks, we asked them to rate the confidence in their performance or to indicate on a scale presented on the tablet the direction and magnitude of their temporal error (whether they arrived too early or too late at the target).

In the confidence self‐evaluation, participants were asked to estimate their performance similarly to the method used in Experiment 1. They were asked to judge whether their performance on that trial was better or worse than the overall performance.

In the temporal error self‐evaluation, participants were asked to indicate their estimated temporal error, by placing the pen somewhere on the blue line that was presented after each trial (Figure 6). The midpoint of the line indicated correct performance. If participants estimated that they arrived too early to the target, they had to place the pen left of the midpoint. If instead they estimated that they arrived too late, they had to place the pen right of the midpoint. The distance from the scale midpoint indicated the magnitude of the temporal error.

Before the experiment, participants completed a training session of 30 trials. During the training session, feedback about their temporal performance was presented after each trial. They were also familiarized with the scale for providing their temporal error estimations and recalibrated their temporal error estimates to the length of the scale used to estimate the error. In each condition of each experiment, participants completed 40 trials, yielding 320 trials in total (three distances in the movement task of Experiment 2 and the keypress task of Experiment 3, for each of the two self‐evaluation tasks). The order of the response method and self‐evaluation tasks was counterbalanced across participants.

## 5 RESULTS AND DISCUSSION

**FIGURE 7:**
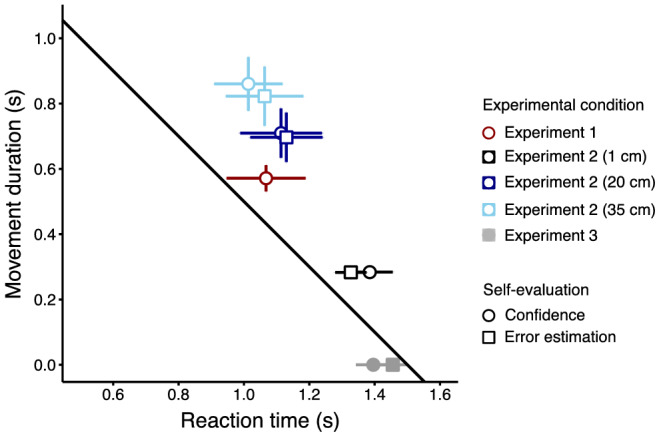
Relationship between movement duration and reaction time in Experiments 2 and 3. Reaction time and movement duration are averaged across participants. Performance in the hand movement task of Experiment 2 is shown in open symbols, in different shades of blue for the three target distances. Performance in the keypress task of Experiment 3 is shown in grey‐filled symbols, with a movement duration set to zero by definition. The diagonal line indicates the trade‐off between reaction time and movement duration that is compatible with the temporal interval that participants had to synchronize to (1.5 s). For reference, the red symbol shows the results from the Experiment 1, for the condition where the interval was also 1.5 s, averaged across different target sizes. Different symbol shapes represent different self‐evaluation methods (confidence for circles and error estimation for squares). Error bars indicate the standard errors of the mean between participants

To assess performance in Experiment 2, we averaged movement duration and reaction time, separately for each target distance and self‐evaluation method. We performed the same analysis for Experiment 3, averaging reaction time separately for each self‐evaluation method. The averaged movement duration is plotted against the reaction time in Figure 7, separately for each distance, response task and the two self‐evaluation tasks. In Experiment 2, participants arrived at the target too late, and the error increased with the target distance. These results replicate the ones we obtained in Experiment 1. The different self‐evaluation methods did not affect performance in the timed motor tasks (circles and squares overlap for the same distance condition in Figure 7).

### 5.1 Confidence judgements are related to deviations from median reaction time but not movement duration

**FIGURE 8:**
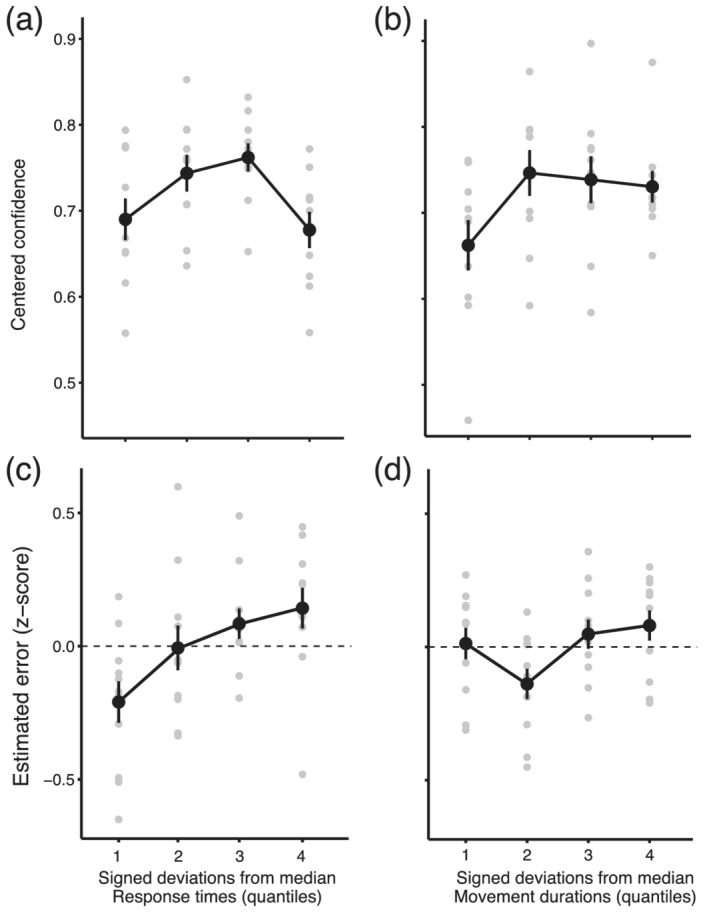
Relationship of confidence and estimated error judgements to reaction times and movement durations in Experiment 2. (a) Average proportion of responses ‘performance better than average’ (centred, Loftus & Masson, 1994) is plotted against signed deviation from median reaction time (quartiles). Confidence was greater when the reaction time on a given trial was closer to the median. (b) Average proportion of responses ‘performance better than average’ (centred) is plotted against signed deviations from median movement durations (quartiles). There was no systematic relationship between movement duration and confidence. (c) Average standardized estimated temporal error is plotted against signed deviations from median reaction time (quartiles). There was a positive relationship between the deviations in reaction time and movement duration. The greater the deviation from the median, the greater the estimated temporal error. (d) Average standardized estimated temporal error plotted against signed deviations from median movement durations (quartiles). There was again a positive relationship between the deviations in movement duration and the standardized estimated error. Performance was averaged across the three target distances. Error bars indicate standard errors of the mean across participants, and grey points show individual data. Deviations from median reaction times and movement durations were averaged across the four quartiles of their distributions, for the purpose of data visualization

We then assessed the relationship between confidence judgements and different components of the movement execution. For this purpose, we calculated trial‐to‐trial deviations in reaction time and movement duration away from their medians, separately for each participant and target distance. For reaction times (Figure 8a), deviations from the median indicate how much the reaction time on a given trial deviates from the planned reaction time. There was a negative relationship between absolute deviations in reaction time and confidence. The smaller the absolute deviation from the median reaction time (closer the reaction time was to the planned reaction time), the greater was the confidence in the performance. Similarly for movement duration (Figure 8b), deviations from the median indicates how much the movement duration on a given trial deviates from the planned movement duration. Interestingly, there were no relationships between confidence and planned movement duration.

To quantify the effect of deviations from median reaction time and movement duration, we conducted a generalized linear mixed‐effect model, with signed and squared deviations from median reaction time and movement duration and the target distance as predictors and binary confidence judgements as the dependent variable. We included as random structure intercept and slope for the target distance at the level of participant. The only significant effect was the squared deviation from reaction time (Wald Chi‐square(1) = 6.732, *p* < .01). The more reaction time on a given trial deviated from the median reaction time, the greater was the likelihood that performance on that trial will be chosen as less confident than average performance (*b* = −1.40, SE = 0.54, *z* = −2.6, *p* < .01).

Correcting for effects of learning or fatigue did not change the outcome of the analysis (significant effect of the squared deviations from the median reaction time only, Wald Chi‐square(1) = 5.1769, *p* < .05).

### 5.2 Temporal error estimations are related to deviations from median reaction time and movement duration

In order to quantify the performance in the explicit estimation of temporal error, we tested whether estimated error was related to the actual temporal error (Figure 8c,d). We first standardized estimated temporal error for each participant, by calculating *z* score of each estimate relative to the mean and standard deviation for a particular distance condition. To test whether deviations from median reaction time and movement duration contributed to estimation of temporal error, we calculated signed deviations from median reaction time and movement duration for each participant and distance. We found positive relationships between the estimated errors and both signed deviations from median reaction time and movement duration, indicating that participants genuinely estimated both the direction (too early or too late) and the magnitude of their errors.

We quantified the effect by means of a linear mixed‐effect model, with signed and squared deviations in reaction time and movement duration and the target distance as predictors and estimated error as a dependent variable. We included subjects as a random intercept and a random slope for the distance from the target. There was a significant effect of signed deviations in the reaction time (Wald Chi‐square(1) = 33.744, *p* < .01), movement duration (Wald Chi‐square(1) = 11.17, *p* < .01) and the squared deviations from the median reaction time (Wald Chi‐square(1) = 11.58, *p* < .01). There was a positive relationship between the signed deviations in reaction time (*b* = 0.762, SE = 0.132, *t* = 5.809, *p* < .01) and movement duration (*b* = 7.719, SE = 0.231, *t* = 3.344, *p* < .01) and the *z* score of the estimated temporal error. There was also a negative relationship between estimated temporal error and squared deviations from the median reaction time (*b* = −8.940, SE = 0.263, *t* = −3.404, *p* < .01), accounting for a non‐linearity in the relationship (see Figure 8c).

Correcting for learning and fatigue effects did not change the pattern of results (signed deviation from median reaction time: Wald Chi‐square(1) = 23.82, *p* < .01; movement duration: Wald Chi‐square(1) = 7.13, *p* < .01; squared deviation from median reaction time: Wald Chi‐square(1) = 5.83, *p* < .01).

### 5.3 Confidence and temporal error judgements are related to temporal errors

**FIGURE 9:**
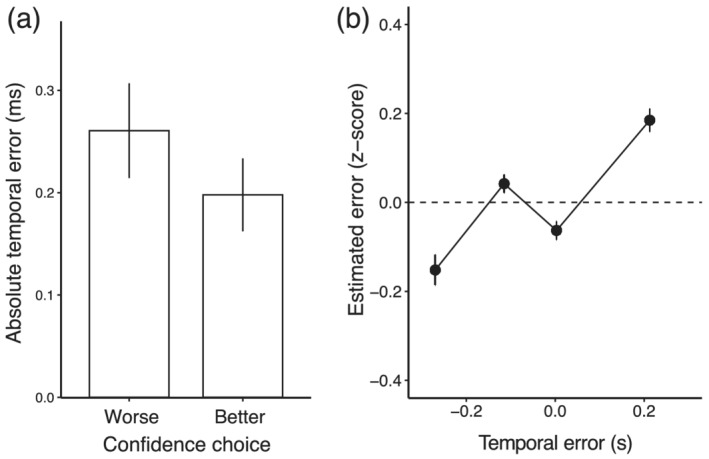
Relationship of confidence and estimated errors to actual errors in Experiment 3. (a) Performance in the confidence self‐evaluation. The average absolute temporal error across participants is plotted as a function of confidence choice (performance on each trial better or worse than average performance). Absolute temporal error was smaller for trials chosen as better than average, indicating metacognitive sensitivity in this task. (b) Performance in the temporal error self‐evaluation. Standardized estimated temporal error is plotted against the actual temporal error. For the purpose of visualization, we binned the temporal error in four quartiles of the temporal error distribution. Each point represents the average standardized estimated error in each quartile, averaged across participants. There was a positive relationship between the actual temporal error and the standardized estimated temporal error. In all plots, error bars indicate standard errors of the mean across participants

The analysis of keypresses in Experiment 3 allows us to verify that performance in the two self‐evaluation tasks is related to the actual performance of the participants. Results for the two self‐evaluation conditions are summarized in Figure 9.

We quantified self‐evaluation judgements by means of two linear mixed‐effect models. To test whether confidence judgements were related to the absolute temporal error, we conducted a generalized linear mixed‐effect model, with binary confidence decision as dependent variable and absolute temporal error as predictor. We also included a random intercept at the level of subject, to account for additional variability. Results showed that the absolute temporal error indeed predicted confidence judgements (Wald Chi‐square(1) = 6.9, *p* < .01). The log ratio of participants choosing a trial as more confident than average increased as the absolute temporal error decreased (*b* = −2.48, SE = 0.94, *z* = −2.64, *p* < .01).

To quantify performance in the temporal error evaluation, we conducted a linear mixed‐effect model, with *z* score of estimated error as dependent variable and actual temporal error as predictor. Participants were included as random intercept, to account for additional variability. As expected, we found an effect of temporal error on estimated temporal error (Wald Chi‐square(1) = 14.83, *p* < .01). Normalized estimated temporal error was positively related to actual temporal error (*b* = 0.720, SE = 0.18, *t* = 3.852, *p* < .01).

## 6 GENERAL DISCUSSION

In the work reported here, we investigated how humans perform and self‐evaluate their action in a sensory‐motor temporal task. In the first experiment, participants synchronized their arm movement with a predictable temporal sequence. After performing the movement, they were asked to judge their confidence in their action by comparing their performance on the current trial to their performance on all previous trials. We varied the temporal interval, size of the target they were aiming at and the target's orientation. In Experiment 2, participants synchronized their movement to a single temporal interval, towards targets that could be at different distances across trials. In Experiment 3, instead of performing hand movement towards the target, participants simply pressed a key to perform their timed action. In Experiments 2 and 3, after participants had performed the temporal task, they were asked to make a confidence estimation or, in other trials, to estimate the magnitude and direction of their temporal errors.

### 6.1 Reaction time and movement duration trade‐off

In Experiment 1, reaction time, movement duration and their trade‐off systematically varied with intervals and target sizes, indicating that our experimental manipulations were successful. Performance was better for longer intervals, presumably because synchronizing with longer intervals offered more time to plan and correct the movement. Furthermore, participants increased slightly their movement durations to aim at targets of smaller sizes. Reaction time was shorter for smaller targets, to compensate for longer movement durations. These results suggest that when planning a movement, both reaction time and movement duration are taken into account. However, participants mostly varied their reaction time, keeping movement duration brief and relatively constant across conditions. The isochrony, or tendency to keep movement duration constant for different distances, is a well‐known principle in motor control (Engelbrecht, 2001; Freund, 1986; Viviani & Flash, 1995; Wolpert, 1997). Similar to the isochrony for spatial distance, our results are consistent with a tendency to keep movement durations constant across temporal intervals.

Humans have an ability to use their knowledge of uncertainty about their perception and movement in space (Trommershäuser et al., 2003) and time (Hudson et al., 2008) to properly plan and execute actions. However, the knowledge can be limited or biased (Maloney & Zhang, 2010; Mamassian, 2008). The trade‐off we observed suggests that participants could have incorrect representations of the uncertainties of their reaction time and movement duration. More specifically, participants systematically chose longer reaction times than movement duration, in spite of the fact that motor variability scaled faster with reaction time than with movement duration (coefficients of variation for reaction time were larger than for movement duration, *t*(199) = 6.842, *p* < .01). Why would participants adopt this strategy? Participants could aim at minimizing the perceived costs of their action, if moving for a shorter time, rather than performing slow and long movements, is less costly (Cos et al., 2012; Cos et al., 2014; Dean et al., 2007). Alternatively, participants could perceive the movement onset as more controllable than the movement duration. Recent work provided evidence that flexible sensorimotor timing is achieved by populations of neurons whose interactions over time can be modelled as a dynamical system (Fetz, 1992; Remington, Egger, et al., 2018; Shenoy et al., 2013). In this framework, complex patterns of neural activity can be described as changes in neural states or positions defined in the coordinate system (state space) in which axes correspond to activities of neurons. These changes of the neural states form neural trajectories, which can be complex and non‐linear. Because state space does not change on small time scales, the adjustments needed for adequate movement planning and execution are made either by external input to the state space or by adjustments of initial conditions preceding the movement (Shenoy et al., 2013). The pattern of behaviour we observed can be understood in the context of constraints imposed by this design. Adjustments along trajectories require knowledge about dynamics around the nonlinear movement trajectory, and small adjustments could have large effects on the movement (Remington, Egger, et al., 2018; Shenoy et al., 2013). During a closed‐loop, continuous movement as in our task, these adjustments become even more complex because in addition to the initial conditions and the external input, the inputs to the network are continuously coming from proprioceptive and visual feedback (Conditt & Mussa‐Ivaldi, 1999; Leib et al., 2017). Therefore, adjusting the reaction time on a trial‐to‐trial basis, keeping the movement duration constant and performing stereotypical movements across the conditions could be an advantageous strategy.

In Experiment 2, we found that participants systematically arrived at the target too late. The further away the target was from participants, the greater was the error (Figure 7). These results are in agreement with the results of the Experiment 1, because participants overall arrived at the target too late. In contrast, when movement duration was minimized as this was the case in the interval reproduction task of Experiment 3, no systematic temporal bias was observed (filled symbols in Figure 7). This suggests that in Experiment 1, the observed bias of the response times of participants resulted from an underestimation of movement duration that corresponded to the longer movement needed to reach the far targets. Due to the signal dependent noise in movement execution, increasing the speed of the movement results in greater spatial end‐point variability. Trading off the spatial precision for the temporal accuracy could yield the observed pattern of results. However, in order to account for differences in spatial precision with movement duration, we did increase the stimulus size with distance, which made the spatial performance for stimuli at different distances comparable. Further work is needed to specifically disentangle the different sources of the observed bias.

### 6.2 Accuracy and confidence can be dissociated for timing an action

In Experiment 1, participants used different cues to estimate their confidence about their performance in a temporal synchronization task. Both the spatial and temporal errors were affected by the orientation of the target, and performance was better if the target was oriented horizontally. However, confidence did not depend on target orientation. Therefore, participants did not take into account all the parameters that were affecting their performance. In agreement with previous findings showing that humans do not always have an accurate representation of their spatial (Wu et al., 2006; Zhang et al., 2013; Zhang et al., 2015) or temporal (Mamassian, 2008) motor uncertainty, we observed that participants also failed to properly monitor the effect of target shape on both spatial and temporal performance.

We also found that interval duration affected both performance in the timed action and participants' estimated confidence but in opposite directions. Performance was better for longer intervals, but participants were less confident on those trials. Because shorter intervals are encoded with less uncertainty (Gibbon, 1977; Jazayeri & Shadlen, 2010), it is as if participants were using sensory uncertainty as one of the main cues for estimating confidence in their actions, rather than just motor uncertainty. These findings highlight the complexity of metacognitive judgements that must integrate and compare multiple sources of information (Barthelmé & Mamassian, 2010; de Gardelle & Mamassian, 2014).

### 6.3 Reaction time and movement duration contribute differently to different meta‐cognitive judgements

In Experiment 1, we found that confidence judgements were related to squared deviations from planned movement onset but not movement duration. Detrending reaction time, movement duration and confidence judgements in Experiment 1 revealed linear trends between signed deviations from both movement onset time and movement duration medians, in addition to the squared deviations from the movement onset time. In Experiment 2, we replicated contribution of deviations from planned movement onset, in conditions in which the contribution of sensory uncertainty was minimized, and movement duration and reaction times varied to a greater extent. Furthermore, we found that participants were able to take into account their movement duration when explicitly asked to estimate their temporal error on each trial. Taken together, these findings suggest that information about movement duration can be accessed but is utilized differently for judgements of confidence.

Previous work showed that humans have a limited access to the movement execution once the movement is initiated (Blakemore et al., 2000; Cardoso‐Leite et al., 2010; Frith et al., 2000; Kepecs et al., 2008; MacKinnon & Rothwell, 2000; Riemer et al., 2019; Wolpert, 1997). Furthermore, temporal information might not be explicitly represented during the movement execution but emerge from observed position, velocity and acceleration (Leib et al., 2017). Our results suggest that participants can assess whether their movement duration was long or short compared with the median movement duration (signed deviations) and use that information for self‐evaluation of their performance, but the manner in which they utilize deviations from median reaction time is more intricate. In particular, relationship between deviations from the median reaction time and confidence judgements suggests that participants take into account how different from the *planned* reaction time the performance was on a given trial, and the more the performance deviates from the planned movement onset, the lower the confidence about the performance.

Which information could humans use to evaluate their movement onset time? When estimating temporal errors in a temporal production task by means of two successive keypresses, power in the alpha range after the second keypress is related to the explicit temporal error estimates. Interestingly, the power of the beta oscillations (13–30 Hz) *before* the first keypress was related to this alpha activity but not to actual reproduced duration (Kononowicz & van Wassenhove, 2019). Beta activity is known to reflect inhibition related to motor activity (Engel & Fries, 2010), and it is possible that the read‐out of the motor inhibition before the onset of the movement is used when estimating confidence. In contrast, when explicitly asked to estimate their temporal error, participants are motivated to use different additional source of information (the postmovement alpha) (Charles & Yeung, 2019; Kononowicz & van Wassenhove, 2019; Moran et al., 2015; Yu et al., 2015). Similarly, in the framework of a dynamical system, the confidence estimates could rely on a read‐out of an initial state of the network before the movement onset (Remington, Narain, et al., 2018; Wang et al., 2016). Relationship between the reaction time and confidence judgements we observed suggests that metacognitive system has access to the read‐out of the motor system and that it evaluates this information in the context of the planned action.

Although there was some evidence showing that confidence estimation was related to signed deviations from movement duration median in Experiment 1, this relationship was not replicated in Experiment 2. Why is there a difference in the pattern of results concerning confidence estimation between these two studies? In Experiment 1, different conditions (interval duration, target size and orientation) were interleaved, movement duration medians varied little across the conditions, and there was considerably larger number of trials in total. It is possible that in order to use movement duration as a cue for confidence estimation, more time and evidence is needed, but further work is needed to specifically address this question. In contrast, a robust effect of squared deviations from median reaction time on confidence judgements was found across experiments.

In the interval reproduction task of Experiment 3, we found that both confidence judgements and temporal error estimations were related to actual performance. These results indicate that humans are able to explicitly estimate their performance in an anticipatory timing task, which is an agreement with previous work using similar tasks (Akdoğan & Balci, 2017; Gorea et al., 2010; Kononowicz et al., 2019; Mamassian, 2008). In this reproduction task, movement duration is reduced to a minimum, and the hitting time corresponds almost entirely to the reaction time.

Relating confidence judgements to error estimation has recently become a central subject in meta‐cognition research (Boldt & Yeung, 2015; Charles & Yeung, 2019; Scheffers & Coles, 2000; Yeung & Summerfield, 2012). Recent work suggests that different metacognitive tasks, such as confidence and error monitoring, can be decoded from the same neural activity (Boldt & Yeung, 2015) and rely on the same information (Charles & Yeung, 2019). However, these results are at odds with a recent temporal error monitoring study, in which no relationship between the standard error related negativity and errors in the temporal production task was found (Kononowicz & van Wassenhove, 2019), as well as our findings. While reviewing this literature, it is important to note that in these previous studies, participants are asked to make a self‐evaluation of their performance with a single measure, for example, a confidence rating on a bidirectional scale. In our experiments, we used two distinct self‐evaluation measure: the evaluation of the correctness of their performance on a given trial relative to the overall performance (confidence estimation) and the estimation of the direction and magnitude of the temporal error on each trial. We found that when asked different questions about their performance, participants used different sources of information about the movement execution for their confidence judgements and their explicit error estimation.

We have a final commentary on the method we used to measure confidence. We asked participants to evaluate their performance on a single trial, by comparing it with their estimated average performance. This method is known as the method of single stimuli in perception (Morgan et al., 2000; Nachmias, 2006). We chose this method to probe confidence judgements in an attempt to reduce the effects of known idiosyncratic biases of participants that are known to affect reliability and biases of confidence judgements when confidence ratings are used (Mamassian, 2016). To avoid these idiosyncratic biases, we have used in other works the confidence forced‐choice paradigm where participants have to choose out of two consecutive trials the one they feel they performed better on (Barthelmé & Mamassian, 2009, 2010; de Gardelle & Mamassian, 2014). Unfortunately, it was not feasible to use this paradigm here, because trials in our experiment were rather long. Using the method of single stimuli forces us to be cautious with the interpretation of our results. Given that movement durations varied less than reaction times across conditions, movement duration contributed less to the average performance (notably in Experiment 1), and this could explain why movement duration differently contributed to confidence estimation on individual trials. On the other hand, when estimating the magnitude of the temporal error of the timed action in each trial, participants could have access to the information about their actual movement duration.

In summary, we found that estimating confidence in a temporal sensorimotor task can be biased. In particular, participants used deviations from planned movement onset but not movement duration to estimate confidence in their performance. However, when explicitly asked to estimate their temporal errors on individual trials, participants do take into account information about the two components of the movement execution in a similar manner. Future work should address possible benefits and costs of using different representations when evaluating one's own performance in different tasks.

## CONFLICT OF INTEREST

Authors declare no conflict of interest.

## AUTHOR CONTRIBUTIONS

LJ, PM and JLM designed the study. LJ collected the data and performed the data analysis and interpretation under supervision of PM and JLM. LJ drafted the manuscript, and PM and JLM provided revisions. All authors approved the final version of the manuscript.

### PEER REVIEW

The peer review history for this article is available at https://publons.com/publon/10.1111/ejn.15378.

## Supporting information


**Figure S1.** Temporal errors against presented interval for the two target orientations, shown separately for trials in which movement was finished outside (A, spatial miss) or inside the target (B, spatial hit). Temporal errors are the differences between hitting times (temporal interval between the second target and the moment when participants ended their movement) and interval between the first two stimuli. Positive errors indicate that participants responded too late. Temporal errors are averaged across target sizes. Temporal errors decreased with the interval, reaching bias‐free performance for the longest interval on average. On all panels, error bars represent standard error of the mean across participants, and size of the symbols indicates average number of trials (spatial misses were overall less frequent).
**Figure S2.** Confidence judgments in Experiment 1. (A) The proportion of trials estimated to have better than average performance is plotted against target size. Confidence of participants about their performance increased with the size of the target. (B) The proportion of trials estimated to have better than average performance is plotted against interval duration. Confidence about one's performance was smaller for longer temporal intervals, even though performance was more accurate for these longer intervals. Open symbols indicate performance on trials in which the movement finished outside the target (spatial miss) and filled symbols indicate performance on trials in which movement finished inside the target (spatial hit). Size of the symbols indicates the proportion of trials averaged in that condition. Error bars are standard error of the mean across participants.
**Figure S3.** Average distributions of reaction time, shown separately for each condition in the experiment. In each panel, performance in different interval duration conditions is color coded. Top row shows performance for horizontally and bottom rows for vertically oriented targets. Target size condition is indicated in the top left corner of each panel.
**Figure S4.** Average distributions of movement duration, shown separately for each condition in the experiment. In each panel, performance in different interval duration conditions is color coded. Top row shows performance for horizontally and bottom rows for vertically oriented targets. Target size condition is indicated in the top left corner of each panel.
**Figure S5.** Illustration of exponential fits do (A) reaction time, (B) movement duration and (C) confidence judgements for one participant. For illustration purposes only, data were binned across the trials (normalized) in 50 equally sized bins.
**Table 1.** Median values of fitted coefficients for reaction time, movement duration and confidence.

## Data Availability

All materials and supporting data are available upon a reasonable request to the corresponding author.
