# Supplementary material for: Contrasting contributions of movement onset and duration to self‐evaluation of sensorimotor timing performance
Source: Eur J Neurosci. 2021 Jul 13;54(3):5092–111. doi: 10.1111/ejn.15378 (PMC9291449; doi:10.1111/ejn.15378)
Supplement: Supplementary file 1 — Figure S1. Temporal errors against presented interval for the two target orientations, shown separately for trials in which movement was finished outside (A, spatial miss) or inside the target (B, spatial hit). Temporal errors are the differences between hitting times (temporal interval between the second target and the moment when participants ended their movement) and interval between the first two stimuli. Positive errors indicate that participants responded too late. Temporal errors are averaged across target sizes. Temporal errors decreased with the interval, reaching bias‐free performance for the longest interval on average. On all panels, error bars represent standard error of the mean across participants, and size of the symbols indicates average number of trials (spatial misses were overall less frequent). Figure S2. Confidence judgments in Experiment 1. (A) The proportion of trials estimated to have better than average performance is plotted against target size. Confidence of participants about their performance increased with the size of the target. (B) The proportion of trials estimated to have better than average performance is plotted against interval duration. Confidence about one's performance was smaller for longer temporal intervals, even though performance was more accurate for these longer intervals. Open symbols indicate performance on trials in which the movement finished outside the target (spatial miss) and filled symbols indicate performance on trials in which movement finished inside the target (spatial hit). Size of the symbols indicates the proportion of trials averaged in that condition. Error bars are standard error of the mean across participants. Figure S3. Average distributions of reaction time, shown separately for each condition in the experiment. In each panel, performance in different interval duration conditions is color coded. Top row shows performance for horizontally and bottom rows for vertically oriented targets. Target siz [file EJN-54-5092-s001.pdf]

Confidence and spatial hits and misses

Since number of spatial hits and misses was very different, we represented the average number of trials for each category with different sizes of the symbols.

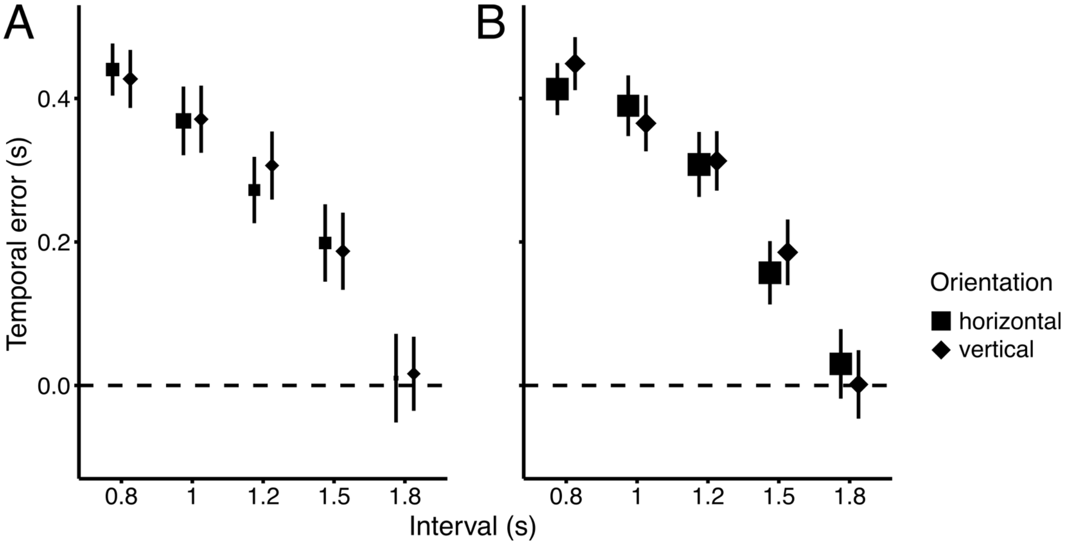

S1. Temporal errors against presented interval for the two target orientations, shown separately for trials in which movement was finished outside (A, spatial miss) or inside the target (B, spatial hit). Temporal errors are the differences between hitting times (temporal interval between the second target and the moment when participants ended their movement) and interval between the first two stimuli. Positive errors indicate that participants responded too late. Temporal errors are averaged across target sizes. Temporal errors decreased with the interval, reaching bias-free performance for the longest interval on average. On all panels, error bars represent standard error of the mean across participants, and size of the symbols indicates average number of trials (spatial misses were overall less frequent).

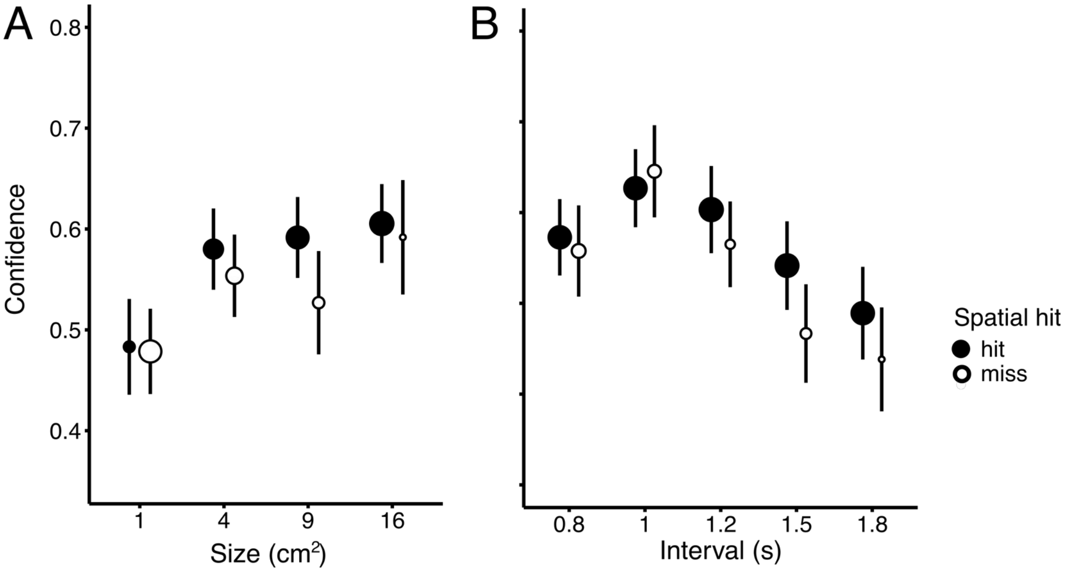

S2. Confidence judgments in Experiment 1. (A) The proportion of trials estimated to have better than average performance is plotted against target size. Confidence of participants about their performance increased with the size of the target. (B) The proportion of trials estimated to have better than average performance is plotted against interval duration. Confidence about one's performance was smaller for longer temporal intervals, even though performance was more accurate for these longer intervals. Open symbols indicate performance on trials in which the movement finished outside the target (spatial miss) and filled symbols indicate performance on trials in which movement finished inside the target (spatial hit). Size of the symbols indicates the proportion of trials averaged in that condition. Error bars are standard error of the mean across participants.

Reaction time and movement duration distributions

Distributions of reaction time and movement duration were averaged by means of the Vincent method (Ratcliff, 1979). For each participant and condition, reaction times and movement durations were ranked, divided and averaged between five quantiles. To obtain average distributions, averaging between participants was done for each of the five quantiles, separately for each condition, and cumulative normal functions were fitted to the average cumulative probability for a different reaction time and movement duration bins. Leftward shifts indicate shorter latencies.

In the figure S3 and S4, the average distributions are shown for each condition in the experiment. Each panel shows distributions for the five interval durations, separately for the two orientations and the four sizes of stimuli.

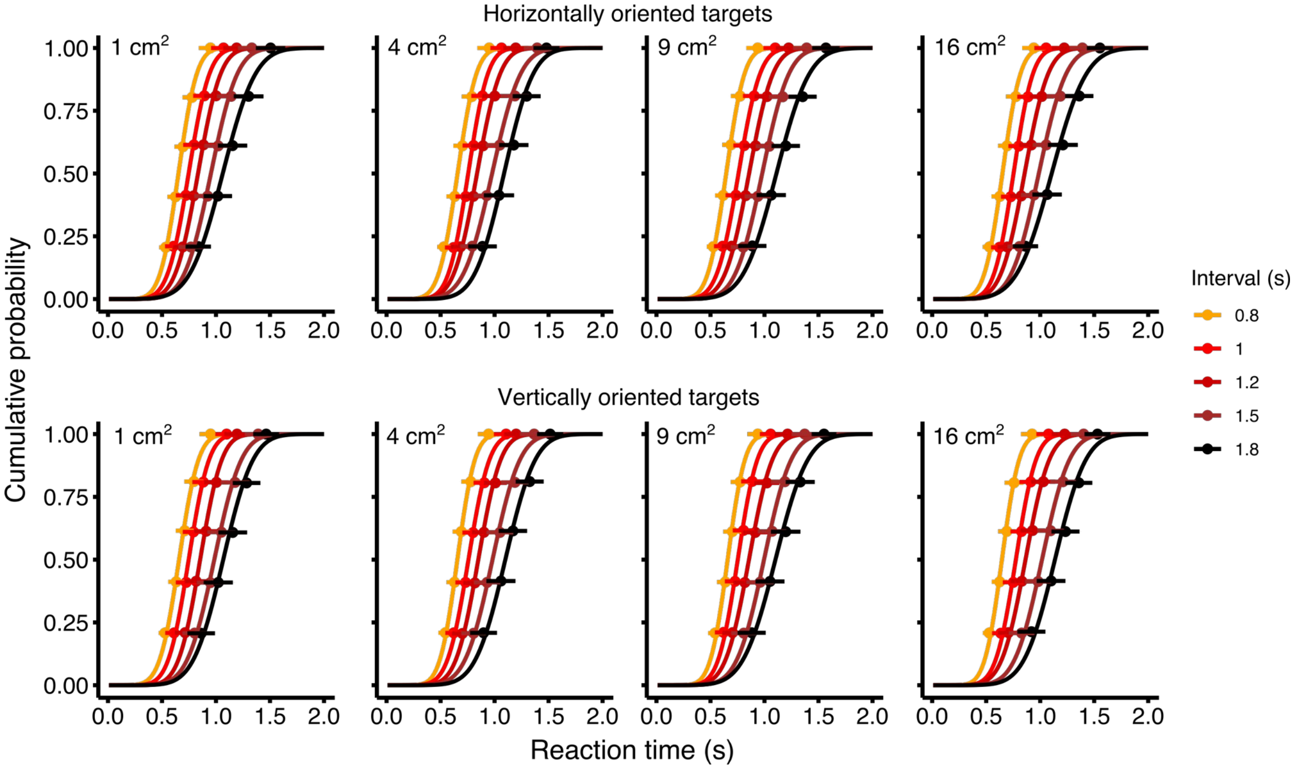

S3. Average distributions of reaction time, shown separately for each condition in the experiment. In each panel, performance in different interval duration conditions is color coded. Top row shows performance for horizontally and bottom rows for vertically oriented targets. Target size condition is indicated in the top left corner of each panel.

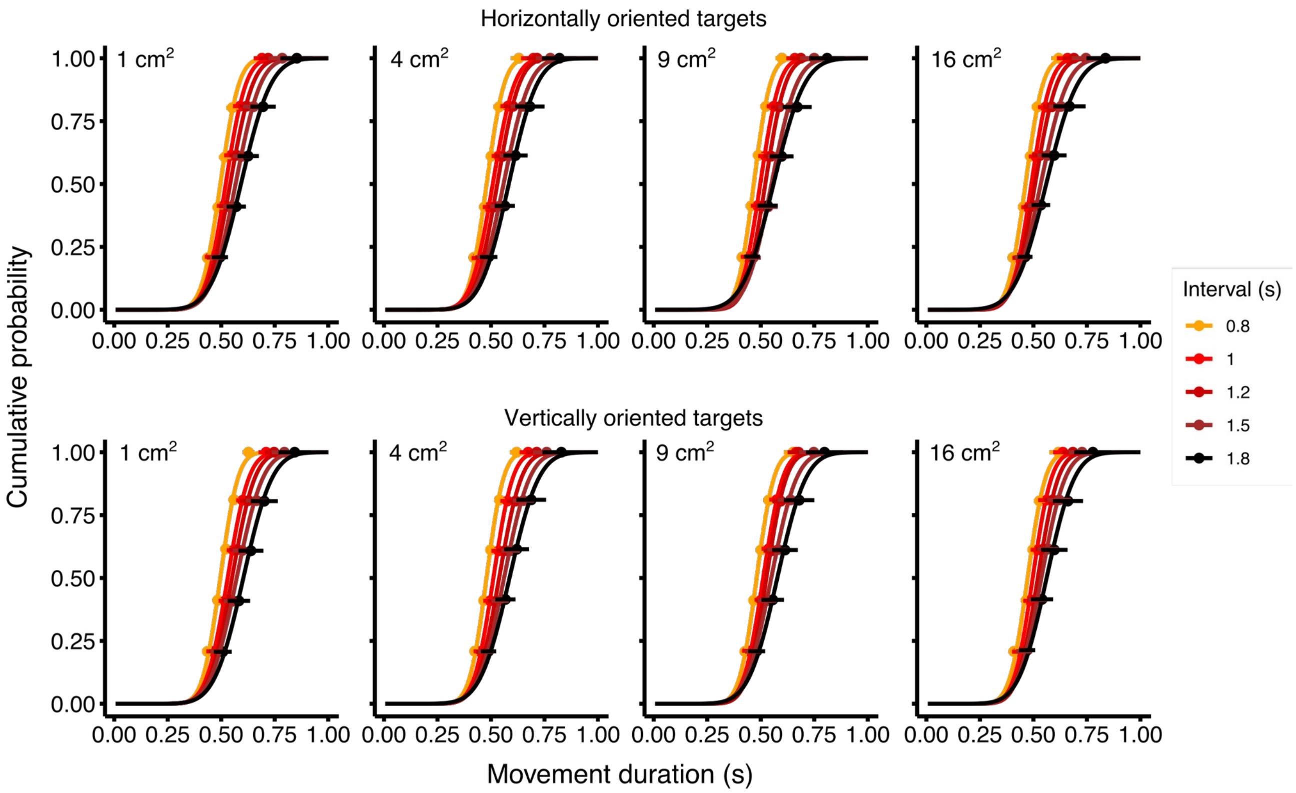

S4. Average distributions of movement duration, shown separately for each condition in the experiment. In each panel, performance in different interval duration conditions is color coded. Top row shows performance for horizontally and bottom rows for vertically oriented targets. Target size condition is indicated in the top left corner of each panel.

Exponential fits

In order to assess and correct for effects of learning or fatigue over the course of the experiment, we fitted individual reaction times, movement durations and confidence judgements to an exponential function:

$$y = b_1 + b_2 \cdot e^{-b_3 x}$$

Then, to obtain detrended values, we calculated residuals between the observed data and obtained fits. For reaction time and movement duration, these residuals were used in the subsequent analysis. For confidence judgements we additionally scaled residuals to the 0 to 1 range. We used *lsqcurvefit* for non-linear fits of reaction time and movement duration and maximum likelihood estimation (*fminsearchcon*) for the binary confidence judgements as implemented in Matlab.

Example fits are shown for one participant in Figure S5, and median values of parameters are shown in Table 1. For illustration purposes only, data were binned across the trials (normalized) in 50 equally sized bins. The analyses were done on single trial data.

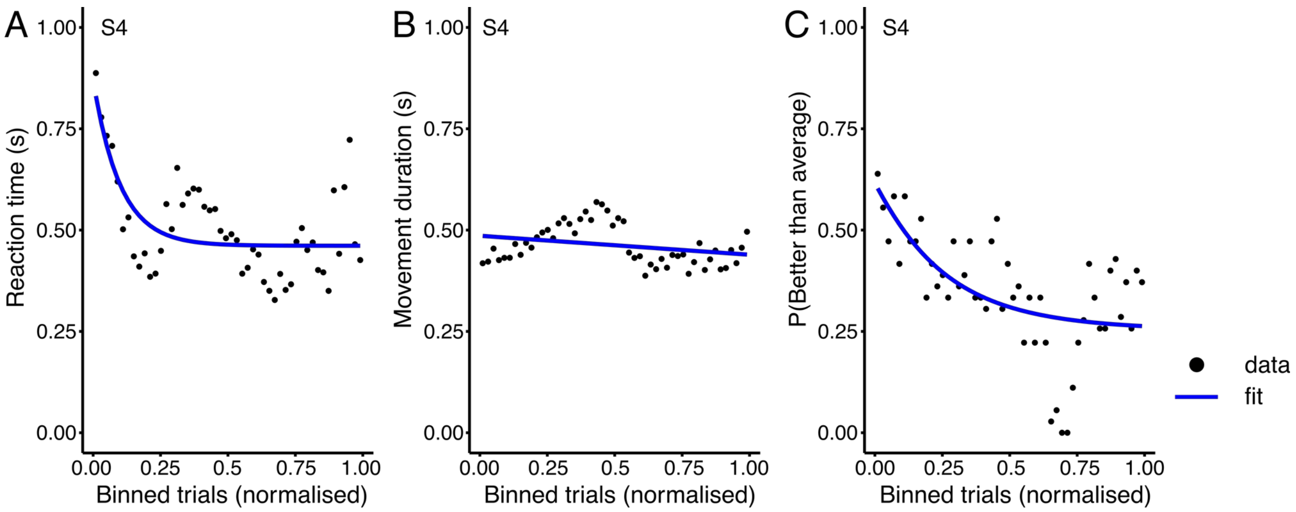

S5. Illustration of exponential fits do (A) reaction time, (B) movement duration and (C) confidence judgements for one participant. For illustration purposes only, data were binned across the trials (normalized) in 50 equally sized bins.

	**Coefficients** Median (MAD)		
**Variable**	b1	b2	b3
Reaction time	1.1 (0.7)	-0.19 (0.9)	-1.7 (2.47)
Movement duration	0.53 (0.13)	0.116 (0.11)	-7.8 (2.8)
Confidence	-0.25 (1.7)	0.02 (2.3)	-6.6 (8)

Table 1. Median values of fitted coefficients for reaction time, movement duration and confidence.

Supplementary references

Ratcliff, R. (1979). Group reaction time distributions and an analysis of distribution statistics. *Psychological Bulletin & Review*, **86**(3), 446.
